# Singing in and out of COVID

**DOI:** 10.1111/resp.14424

**Published:** 2022-11-30

**Authors:** Debra Jean Phyland

**Affiliations:** ^1^ Department Otolaryngology Head & Neck Surgery, Monash Health Clayton Victoria Australia; ^2^ Faculty of Medicine, Nursing and Health Sciences Monash University Clayton Victoria Australia

**Keywords:** COVID‐19, hoarseness, larynx, occupational health, singing, voice

Never has singing health been more threatened in the 21st century than over this time of global pandemic. COVID‐19 has wreaked havoc on the mental health and livelihoods of occupational voice‐users (OVU) and performers in particular.^1^, ^2^, ^3^, ^4^, ^5^ Aside from the obviously devastating social, economic and wellbeing impacts of not being able to perform due to virus transmission risk, and associated lockdowns and lifestyle changes, COVID‐19's proclivity for entry and passage through the unified airway and symptom complex has also directly jeopardized vocal health. The airway implications of the acute phase of infection, and of course potential effects of intubation and mechanical ventilation^6^ are well recognized but even in mild–moderate cases there are also frequent lingering effects and indeed even long‐term sequelae that pose additional threats to the performer.^7^, ^8^, ^9^, ^10^ Arguably comparative to other occupations, professional singers have been dealt the worst ‘blow’ to their livelihood by the COVID‐19 onslaught.

Singing is both a physical and psychological activity involving artistic expression and athleticism. Since the physical act of voice production relies on integrity and balance between the three key components of power (respiration), source (phonation) and filter (resonance), any compromise to the form and/or function of the vocal tract can hamper the vocal output. Although it seems that some strains may have a propensity for different parts of the airway/vocal tract, with Omicron variant proposed to most affect the larynx/pharynx,^11^, ^12^ COVID‐19 symptoms can occur across all three voice components.

From a *breathing* perspective, greater air volumes and more precise respiratory/phonatory coordination and subglottal pressure control are required for singing than for speech and most other physical activities‐ thus impaired pulmonary function, airway irritability and respiratory muscle weakness due to COVID‐19 infection will negatively impact respiratory drive and kinematics for vocal dynamics and endurance. For hospitalized and severe cases, symptoms commonly persist for many weeks or months as part of the Long COVID‐19 profile^12^ but respiratory sequelae may occur even following infections in persons without severe symptoms,^8^ proving also problematic to the resumption of pre‐morbid singing activity. While mild or moderate reductions in respiratory function may not be greatly impactful for the average person, they can be devastating to the singer and the difference between being able to sing or not.

*Laryngeal* impairment can also be acute or chronic impacting on voice, throat sensation, swallowing and breathing, whether due to the virus itself or the management. Specifically, voice disorders due to vocal fold oedema and inflammation, sensory neuropathy and post‐viral neuropathies are common, corresponding to inflammatory and neurotropic virus mechanisms.^9^ Even in only mild–moderate COVID infections, 1/4 of people have been noted to be still dysphonic some weeks later which is especially problematic for the singer.^10^ Lechien et al. suggest persisting dysphonia may also relate to, or be further compounded by, reflux secondary to the stress of infection whereby the associated autonomic nervous system dysfunction induces increased oesophageal sphincter relaxation.^9^

According to my observations over the pandemic period, in comparison to prior times, presentations of treatment‐seeking singers with acute phonotraumatic lesions, irritable larynx syndrome, vocal paresis, chronic cough, odynophonia, muscle tension dysphonia and related vocal fatigue syndromes have been disproportionately higher in laryngology clinics, many of which have been attributed to COVID. This increase in voice concerns among singers is no doubt partly due to the heightened awareness of vocal health and increased stakes for them over other voice‐user groups but one could speculate this may also be due to a change in vocal health and physiological response to vocal load. Interestingly in contrast, although cases of functional airway obstruction disorders (inducible laryngeal obstruction, EDAC and dysfunctional breathing) have also increased over the pandemic,^13^, ^14^ we have seldom seen singers with these conditions.

The third essential component of voice production, *resonance*, relies on patency of the airway and so tissue inflammation and congestion of the resonators (for e.g., oral and sinonasal cavities, laryngo‐pharynx and chest) can substantially alter the harmonics and airway resistance to phonation for singing. Although a common acute presentation,^15^ the prevalence and duration of persistent sinonasal symptoms with COVID‐19 is variable and again may depend on the acquired variant profile.^12^ Albeit anecdotal, in my voice consultancy work with theatre performers, resonance has been regularly observed to remain altered in singers post‐COVID and well after other symptoms have abated. This has impeded voice quality, ease of production and frequently contributed to vocal fatigue and throat irritation, such that performers have needed reduced performance regimes, longer recovery times or graduated return to work schedules in order to meet vocal demands, despite being seemingly otherwise well.

Singers are vocal athletes and their capacity to undertake the physical activity of singing after COVID can be reduced not just due the local effects of the virus, but other systemic or generalized effects. Chronic fatigue in particular can be highly problematic for performers, especially for those with high vocal loads and concurrent physical demands (e.g., musical theatre singers). In addition, the management of COVID‐19 symptoms may itself be troublesome for voice. Post‐intubation injuries have been well described including laryngeal scarring, subglottal stenosis, arytenoid dislocation and vocal fold trauma^6^ and these can have a highly deleterious impact on voice. Perhaps less recognized are the potentially negative laryngeal effects of inhaled corticosteroid sprays when used for airway inflammation/irritability. Although their administration is recommended for COVID‐related dyspnoea (and well‐established for asthma control), dysphonia is a common local side‐effect of many inhalers significantly hampering source characteristics and therefore voice quality for singers, albeit reversible.^10^ In addition, performers' self‐management strategies to prevent cough, dry up nasal secretions, or fast track resolution of dysphonia can include non‐judicious use of oral steroids or some creative homebrews, which can hinder voice recovery.

Akin to the sports medicine field,^16^ guidelines regarding the timing and approach for return to singing for performers after COVID‐19 require careful consideration of the psychologic and physiologic impacts of the virus, performance capacity and application of targeted rehabilitation programs to optimize fitness.^17^ On a brighter note however, one could postulate that singers might have an advantage over non‐singers when recovering from COVID‐19 infection. Although larger scale studies are required to provide higher‐level evidence of the exact physiologic mechanisms, singing has been shown to offer substantial benefits to immunity, lung health, wellbeing and perceived quality of life in healthy individuals and those with neurological or respiratory diseases. Specifically benefits have been associated with neurotransmitter and hormonal changes, including the upregulation of oxytocin, immunoglobulin A and endorphins, increased respiratory muscle strength, mucociliary airway clearance and optimized breathing modes.^18^, ^19^ Preliminary data regarding the positive role of singing rehabilitation in Long COVID‐19^20^ on pulmonary function also suggests singing is a worthy pursuit for all recovering from COVID and indeed the door to improved wellbeing in these challenging times for all those who find joy in singing (Video 1).

**VIDEO 1:** Singer's larynx 4 weeks post‐COVID

## CONFLICT OF INTEREST

None declared.
